# Therapeutic Angiogenesis of Chinese Herbal Medicines in Ischemic Heart Disease: A Review

**DOI:** 10.3389/fphar.2018.00428

**Published:** 2018-04-26

**Authors:** Dongqing Guo, Colin E. Murdoch, Tianhua Liu, Jia Qu, Shihong Jiao, Yong Wang, Wei Wang, Xing Chen

**Affiliations:** ^1^School of Life Sciences, Beijing University of Chinese Medicine, Beijing, China; ^2^Division of Molecular and Clinical Medicine, School of Medicine, University of Dundee, Dundee, United Kingdom; ^3^School of Traditional Chinese Medicine, Beijing University of Chinese Medicine, Beijing, China; ^4^School of Information and Control Engineering, China University of Mining and Technology, Xuzhou, China

**Keywords:** ischemic heart disease, treatment status, Chinese herbal medicines, therapeutic angiogenesis, targets

## Abstract

Ischemic heart disease (IHD) is one of the primary causes of death around the world. Therapeutic angiogenesis is a promising innovative approach for treating IHD, improving cardiac function by promoting blood perfusion to the ischemic myocardium. This treatment is especially important for targeting patients that are unable to undergo angioplasty or bypass surgery. Chinese herbal medicines have been used for more than 2,500 years and they play an important role alongside contemporary medicines in China. Growing evidence in animal models show Chinese herbal medicines can provide therapeutic effect on IHD by targeting angiogenesis. Identifying the mechanism in which Chinese herbal medicines can promote angiogenesis in IHD is a major topic in the field of traditional Chinese medicine, and has the potential for advancing therapeutic treatment. This review summarizes the progression of research and highlights potential pro-angiogenic mechanisms of Chinese herbal medicines in IHD. In addition, an outline of the limitations of Chinese herbal medicines and challenges they face will be presented.

## Introduction

Ischemic heart disease (IHD), also called coronary artery disease (CAD) (Jaganathan et al., 2014), refers to the condition of stenosis or obstruction of the lumen of arteries and inadequate blood supply to the myocardium leading to myocardial ischemia (MI), hypoxia or necrosis. Clinically, IHD includes asymptomatic MI, angina, myocardial infarction, ischemic heart failure and sudden cardiac death (Wong, 2014). IHD is reported to be the leading reason of disability and death worldwide which caused 8.9 million deaths in 2015, resulting in a large economic burden to the medical community (GBD 2015 Mortality and Causes of Death Collaborators, 2016). Although several risk factors including high blood pressure, smoking, diabetes, lack of exercise, obesity and high blood cholesterol have been identified (Charlson et al., 2013; Mehta et al., 2015), the therapeutic effects are still limited. Therefore, new treatment strategies mitigating the deleterious effects of IHD are being explored intensively. Growing evidence suggest the potential pro-angiogenic benefits of Chinese herbal medicines in IHD. This review will summarize the status and process of the pro-angiogenic roles and mechanism of Chinese herbal medicines in IHD. In addition, the limitations and future challenges will be highlighted.

## Methodology

The United States National Library of Medicine, National Institutes of Health (NIH) database^1^ was searched with the terms “IHD” or “therapeutic angiogenesis” or “Chinese herbal medicines” in the “title/abstract” field. Related articles were chosen manually before 31st December, 2017. All articles which had an abstract available were included. No language restrictions were applied.

## Treatment Status of Ischemic Heart Disease

The front line clinical treatments for IHD include drug therapy, percutaneous coronary intervention (PCI) and coronary artery bypass grafts (CABGs). There are a number of pharmaceutical therapies which are widely used in IHD including nitroglycerin (Boden et al., 2015), statins (Gutierrez et al., 2012), β-blockers (Bauters et al., 2014), and calcium channel blockers (Conti, 2011) but their beneficial effects are limited. In some cases symptoms are still are not significantly improved even upon maximal dosage of drug therapy (Pogosova et al., 2016). PCI is a minimal invasive procedure to treat stenosis of the coronary arteries. A deflated balloon catheter is advanced into an obstructed artery via the saphenous or radial artery. Inflation of the balloon reverses the narrowing of the blood vessel (Osorio et al., 2017). Alternatively, stents are placed in the narrowed artery to ensure blood vessels remain dilated. PCI is restricted to patients whose angiography do not show arteries <1.5 mm in diameter or display diffusely diseased saphenous vein grafts (Montalescot et al., 2014). CABG is a surgical strategy to improve the coronary perfusion and used to treat patients who have severe IHD. During this process, sections of saphenous vein and radial artery are usually used as the vascular bridge. The blood bypasses the lesion of coronary artery and flows to the ischemic myocardium through the vascular bridge. However, the patency of the grafted artery or vein depends on the patient’s vascular status and is likely to be atherosclerotic similar to the coronary vessels. Furthermore, due to the invasive nature it is not a viable procedure in elderly patients, e.g., octogenarians and above (Ohki et al., 2002). Approximately 12% of patients with IHD are unsuited to the classical PCI or CABG treatment (Tandar et al., 2002). This is due to diffuse disease, chronic total occlusion, multiple stenosis, poor targets and severe comorbidities (Mitsos et al., 2012). Therapeutic angiogenesis has attracted great interest in targeting patients that are not viable for the current treatment. Increasing blood supply to the ischemic zone is fundamental to the treatment of IHD. Therefore, therapeutic angiogenesis is the promising approach aimed at returning cardiac function (Sasaki et al., 2002; Koneru et al., 2008). The applications of growth factor therapy, gene therapy and stem cell therapy are becoming more widely used.

Recent findings have indicated that Chinese herbal medicines may be effective in the treatment of IHD by promoting angiogenesis. Focus has turned to pre-clinical investigations to understand the mechanism in which Chinese herbal medicines can induce angiogenesis in IHD. In this review, we summarize current data on the pro-angiogenic role of Chinese herbal medicines in IHD. The review focuses on the research process and prospects of traditional Chinese herbal medicines in therapeutic angiogenesis for IHD.

## Basic Mechanisms of Angiogenesis

Three important processes are required for the development of the vascular system, vascular network formation during embryonic development (vasculogenesis) (Schulte, 1914), microvascular formation (angiogenesis) (Ausprunk and Folkman, 1977; Carmeliet, 2000) and the formation of larger arteries and vessels (arteriogenesis) (Heil et al., 2006). Neovascularization is a term used to describe vessel development (Freedman and Isner, 2001).

**FIGURE 1:**
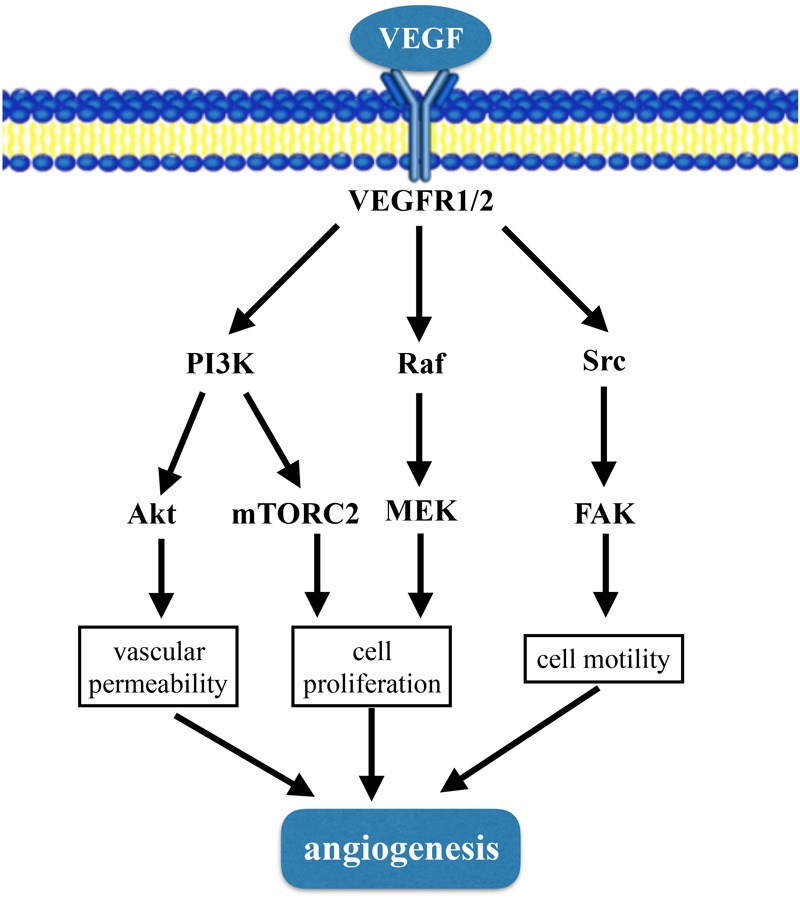
VEGF signaling pathway involved in angiogenesis.

Angiogenesis refers to the development of new blood vessels from pre-existing capillaries (Angulo et al., 2011). The process of angiogenesis includes degradation of vascular basement membrane; proliferation and migration of endothelial cells; lumen structure and vascular network formation (Ren et al., 2014). The mechanism of angiogenesis is complicated and tightly regulation of different cell types, such as endothelial cells, smooth muscle cells, and inflammatory cells. Vascular endothelial cells have the ability of rapid proliferation, migration and differentiation in response to physiological stimulation (Folkman, 1995b). There are many molecules involved in the regulation of angiogenesis, including vascular endothelial growth factor (VEGF), fibroblast growth factor (FGF), transforming growth factor (TGF), tumor necrosis factor (TNF) and angiopoietin-2, etc. VEGF is the most studied, and a large number of pre-clinical experiments have proved that it is effective in promoting angiogenesis. VEGF can activate a plethora of downstream pathways including PI3K-Akt/mTORC2 pathway, Raf-MEK-MAPK pathway and Src-FAK pathway (Matsumoto and Claesson-Welsh, 2001; Claesson-Welsh, 2016) (**Figure 1**). Tissue hypoxia or inflammation is major stimuli of angiogenesis. In settings where angiogenesis is deficient administration of recombinant proteins, or gene and stem cells therapies can enhance the ability for angiogenesis to occur.

## Ischemic Heart Disease and Angiogenesis

### Angiogenic Growth Factor Therapy

Ischemic heart disease is caused by the stenosis or obstruction of coronary blood vessels denying oxygen to the heart muscle. Promoting the heart to generate new blood vessels through angiogenesis provides a potential to combat IHD. Over the last few decades this has been attempted mainly through the delivering cytokines to increase blood perfusion in the ischemic region and ultimately reverse the disease (Cooke and Losordo, 2015). Traditional cytokine-based therapies, such as VEGF, FGF, platelet-derived growth factor (PDGF) and angiopoietins, have shown theoretical and experimental promises for treating ischemic diseases (Harada et al., 1996; Atluri and Woo, 2008).

#### Vascular Endothelial Growth Factors

To date, VEGF is a recognized regulator of angiogenesis and is an endothelial specific mitogen for IHD. The VEGF family comprises of different isoforms, VEGF-A, VEGF-B, VEGF-C, VEGF-D, and placental growth factor (PIGF) (Neufeld et al., 1999). VEGF frequently refers to VEGF-A. VEGF-A contributes to angiogenesis, vasculogenesis, and vascular homeostasis. In contrast to VEGF-A, VEGF-B can’t regulate angiogenesis and it functions in the muscle fatty acids uptake (Mehlem et al., 2016). In addition, VEGF-C and VEGF-D regulate the lymph system and adipose tissue inflammation (Karaman et al., 2015). PIGF is synthesized mainly by trophoblast cells and can bind to tyrosinase receptors in trophoblast cells and vascular endothelial cells. PIGF mainly affects the function of trophoblast cells through autocrine action, and influences the process of vascular growth through paracrine action (Cao et al., 1997). The receptors of VEGFs include VEGFR1 (Flt1), VEGFR2 (Flk1), and VEGFR3. VEGF can promote the proliferation, migration, and angiogenesis of endothelial cells by promoting the mitochondria function (Guo et al., 2017). It was shown in porcine coronary occlusion model that VEGF treatment increased blood flow and recovery of cardiac function (Harada et al., 1996). Intracoronary VEGF treatment enhanced the development of small coronary arteries supplying ischemic myocardium in dogs (Banai et al., 1994). However, there are many limitations in the administration of VEGF. VEGF may induce the progression of atherosclerotic plaques (Celletti et al., 2001). What’s worse, VEGF over-loading has the potential to cause cancer (Hanahan and Folkman, 1996), diabetic retinopathy (Aiello et al., 1994), and rheumatoid arthritis (Folkman, 1995a).

#### Fibroblastic Growth Factors

Fibroblastic growth factors are another important growth factors in angiogenesis, wound healing and embryonic development. The FGFs are heparin-binding proteins and interactions with cell surface-associated heparin sulfated proteoglycans. FGF-FGFR-1 is the most common signaling pathway. It has been reported that vacation could stimulate the expression of FGF2, followed by phosphorylation of FGFR-1 and promote angiogenesis in microvascular endothelial cells (Sun et al., 2017). Up-regulated FGF2/an early growth response protein 1(EGR1) ameliorated cardiac ischemia and systolic dysfunction (Suda et al., 2017). Endothelial FGFR pathways are beneficial for the IHD. Conditional knock out of Fgfr1 and Fgfr2 in endothelial cells attenuated cardiac function compared to control group in myocardial model (House et al., 2016).

#### Angiopoietins

Angiopoietins are parts of vascular growth factors that play an important role in angiogenesis. Angiopoietin cytokines are involved in regulating microvascular permeability and vascular tension. There are four identified angiopoietins: Ang-1, Ang-2, Ang-3, and Ang-4. It has been reported that Ang-1, circulating Ang-2 and Ang-2/Ang-1 ratio (Ang-2/1) were all increased at ST-segment elevation myocardial infarction patients and were related to the serious extend of myocardial damage (Chen et al., 2012). Ang-4 could inhibit the activity of lipoprotein lipase which could hydrolyze triglyceride, thus Ang-4 may be related to the low risk of CAD (Dewey et al., 2016). Ang-2/Tie2 signaling pathway is involved in angiogenesis in inflammatory cornea (Yan et al., 2017). Ang-1, miR-1, and miR-206 functioned in hypoxic induced myoblasts vascularization (Taylor et al., 2017).

Although angiogenic growth factor therapy attracts wide attention, clinical trials for myocardial angiogenesis have produced unstable effects. It is difficult to titer an effective concentration of VEGF in humans and side-effects easily occur. For example, VEGF could increase the permeability of the blood vessels and cause diabetic retina disease. Although, VEGF has been extensively studied further studies are required to gain better efficacy and safety to enable therapeutic angiogenesis.

### Gene Therapy

Various improvements in biological vector such as viruses and small DNA have improved transmission and expression efficacy of cytokines to enable clinical to promote angiogenesis (Khan et al., 2003). The focus has mainly centered on the use of FGF (FGF1 and FGF2), and VEGF A (VEGF-A165 and VEGF-A121). In preclinical studies, new gene transferring techniques including recombinant adenovirus vectors and liposome complexes have been shown to be effective in promoting angiogenesis for myocardial and vascular diseases (Maione et al., 2001). Recombinant adenovirus, co-expressing antimicrobial peptide (PR39) and adrenomedullin (ADM) were injected into infarcted myocardia and attenuated myocardial injury by promoting angiogenesis (An et al., 2017). However, the robustness and safety of gene therapy really deserve more attention for this to become widely used in clinics.

### Stem Cell Therapy

Stem cell therapy is also a promising option for treating IHD and has beneficial effects via multiple mechanisms (Fukuda et al., 2004). Autologous bone marrow cells, skeletal myoblasts, embryonic stem cells, adult mesenchymal stem cells (MSCs), and adult hematopoietic progenitor cells all have the potential to treat IHD. Autologous bone marrow cells were transplanted into the ischemic myocardium in five male patients. Cardiac function and blood perfusion were improved demonstrating promising potential for this therapy (Yoo et al., 2008). Autologous skeletal myoblast transplantation in Phase I clinical studies demonstrated feasibility in treatment of post infarction myocardial injury after 12 months of follow-up, (Siminiak et al., 2004). Crisostomo et al. (2008) isolated Sprague–Dawley rat hearts in an *ex vivo* Langendorff model and subjected them to ischemia/perfusion treatment. Hearts treated with embryonic stem cells showed better post ischemic recovery (Crisostomo et al., 2008). MSCs with activation of Rap1 conferred cardioprotection against myocardial infarction in rats (Khan et al., 2017). Likewise, endothelial stem cells and MSCs also improve cardiac function in this case via increasing vascular density (Rabbani et al., 2017). Further studies will be required to determine the optimal cell type, dose, transmission method. Moreover, the ability to treatment time after ischemia will be important to develop the effectiveness of the therapy.

### Combined Therapy

Gene therapy and bone marrow MSCs therapy are commonly combined. Qin et al. (2017) constructed recombinant adenovirus vectors 5-hERL-IRES-VEGF (Ad5-EIV) combining reporter gene hERL and therapeutic gene VEGF165. The recombinant adenovirus vector was transfected into MSCs (Ad5-EIV-MSCs). In a rat model of myocardial infarction Ad5-EIV-MSCs were transplanted into the peripheral myocardium, effectively preserving cardiac function (Qin et al., 2017). Another study also indicated that CXCR4-overexpressing MSCs could repair heart tissue post myocardial infarction by promoting angiogenesis and alleviate left ventricle remodeling via paracrine signaling mechanism (Wu et al., 2017).

## Chinese Herbal Medicines Targeting Angiogenesis in Ischemic Heart Disease

Being able to manipulate neovascularization is central to treating IHD. Chinese medicines have a basic view on vascular system for years, such as the concepts of “bloodline and blood-collateral.” Bloodline means larger blood vessels and blood-collateral means the smaller vessels. Traditional Chinese medicine believes that Qi is the most basic substance to make up the human body, and Qi and blood are interlinked. Some scholars have identified that Chinese herbal medicines with the action of activating Qi and blood may function in promoting angiogenesis (Zang et al., 2014). Increasing evidence has shown Chinese herbal medicines may be effective in the treatment of IHD, especially Chinese medicines with the effects of replenishing and activating blood or invigorating and replenishing Qi. Chinese herbal medicine monomers (**Table 1**), formula (**Table 2**) and Chinese patent drugs (**Table 3**) all have shown some benefit in stimulating revascularization, which is driving further studies for ischemic diseases more widely in China.

**Table 1:** Monomers and active components of medicinal plants in ischemic heart disease.

Classification	Name	Source	Models	Targets	Reference
Replenishing and activating blood	Salvianolic acid A	Radix Salvia miltiorrhiza	*In vivo*	JNK/PI3K/Akt↓; EPCs, MSCs↑	Guo et al., 2014; Li et al., 2014; Chen et al., 2016
	Tanshinone IIA	Radix Salvia miltiorrhiza	*In vivo*	VEGF and HIF-1a↑	Xu et al., 2009
	Ferulaic acid	Radix Angelica Sinensis	*In vivo*	VEGF, AKT/mTOR↑	Liu et al., 2009; Zhang Q. et al., 2017
Invigorating and replenishing Qi	Rhodiola	Rhizoma Rhodiolae Kirilowii	*In vivo*	Flt-1, Tie-2, HIF-1a, HIF-1β↑	Li et al., 2005; Gao et al., 2009
	Salidroside	Rhizoma Rhodiolae Kirilowii	*In vivo*	Unknown	Zhang J. et al., 2017
	Astragalosides	Shanxi Astragalus membranaceus	*In vivo*	VEGF and bFGF↑	Yu et al., 2015
Other active components of medicinal plants	Berberine	Berberis and Berberis aristata	*In vivo/in vitro*	miR-29b↑	Zhu et al., 2017
	Puerarin	Radix Puerariae	*In vivo*	VEGF and eNOS↑	Zhang S. et al., 2006
	Extract of *Geum japonicum*	Germ japonicum	*In vivo*	Unknown	Li et al., 2006

**Table 2:** Chinese herbal formula in ischemic heart disease.

Name	Composition	Models	Results/effects	Reference
Danggui Buxue Tang (DBT)	Astragalus, angelica	*In vitro*	Unknown	Lin et al., 2017
Xuefu Zhuyu Tang (XFZYT)	Peach kernel, safflower, angelica, chuanqiong, radix paeoniae rubra	*In vitro*	VEGF-VEGFR2↑	Gao et al., 2012
Buyang Huanwu Tang (BYHWT)	Astragalus, angelica, radix paeoniae rubrathe, peach kernel, safflower, earthworm	*In vivo*	VEGFR2-PI3K/Akt↑	Cui et al., 2015

**Table 3:** Chinese patent drugs in ischemic heart disease.

Name	Composition	Models	Results/effects	Reference
Shexiang Baoxin pills	Musk, ginseng extract, bezoar, cinnamon, storax, borneol toad	*In vivo*	20-HETE, EPCs, and VEGF↑	Huang et al., 2017
QI-SHEN-YI-QI	Astragalus, danshen, panax notoginseng, dalbergia	*In vivo*	VEGF, bFGF and PDGF-B↑; miR-223-3p↓	Zhang et al., 2010; Dai et al., 2016
Tongxinluo	Ginseng, leech, scorpion, centipede, cicada, wood louse insects, red peony root	*In vivo*	PDGF, bFGF, ANG-1, and VEGF↑	Cui et al., 2016
Xuesetong soft capsules	Notoginseng total saponins	*In vivo*	VEGF↑	Zang et al., 2014

### Monomers and Active Components of Medicinal Plants

#### Replenishing and Activating Blood

##### Salvianolic acids

Radix Salvia miltiorrhiza (Danshen) is one of the typical blood-activating and stasis-resolving medicines and it offers therapeutic promise for cardiovascular diseases (Wang et al., 2014). Salvianolic acids are the maximum water extraction from Danshen including salvianolic acid A (Sal A) and salvianolic acid B (Sal B). A meta-analysis of five studies was performed to evaluate the effects of Sal A and Sal B on myocardial infarction rats. And it showed that Sal A and Sal B both increased blood vessel density in MI rats (Yu et al., 2017). Another trial identified the anti-apoptotic effects of Sal A against ischemic reperfusion injury in diabetic rats. Sal A has similar actions as a JNK inhibitor through down-regulating JNK/PI3K/Akt pathway (Chen et al., 2016). Stem cells may be the targets of Sal A and Sal B to improve cardiac function and promote angiogenesis. Li et al. (2014) found that Sal A exerted cardioprotection by increasing capillary density in ischemic rat myocardium detected by CD31 staining. Sal A enhanced the activity of endothelial progenitor cells (EPCs) through SDF-1α/CXCR4 axis (Li et al., 2014). *In vitro* experiments showed that Sal B pretreatment could promote MSC differentiated into endothelial cells. Thus injection of Sal B pretreated MSCs improved myocardial infarction in rats by promoting angiogenesis (Guo et al., 2014). Taken together, these findings all illustrate that salvianolic acids could effectively promote cardioprotection in pre-clinical models of IHDs.

##### Tanshinone IIA

Vascular endothelial growth factor is a pivotal pro-angiogenic factor. The transcriptional factor, HIF-1 enhances VEGF expression in response to hypoxia and plays an important role relating to angiogenesis (Hong et al., 2004). Tanshinone IIA (Tan IIA) is the most abundant diterpene quinone in Danshen. Sodium tanshinone IIA asylate injection is used to treat cardiac infarction in China. Tan IIA elicited significant cardioprotective effects by promoting angiogenesis in MI rat model. Tan IIA improved heart function, reduced infarct size and enhanced VEGF and hypoxia-inducible factor 1alpha (HIF-1) mRNA expression (Xu et al., 2009).

##### Ferulaic acid

Ferulaic acid is the main component of Radix Angelica Sinensis. Bone marrow stromal cells (BM-MSCs) play crucial roles in angiogenesis (Fukuda et al., 2004). *In vitro*, BM-MSCs were pre-treated with sodium ferulate and *n*-butylidenephthalide. Then transplantation of pre-treated BM-MSCs into the ischemic stroke zone promoted VEGF production and angiogenesis by AKT/mTOR signaling pathway (Zhang Q. et al., 2017). Liu et al. (2009) synthetized a compound called acetyl ferulaic isosorbide (AFI) by the esterification of ferulaic acid and isosorbide mononitrate. AFI showed protective effects in myocardial ischemia/reperfusion (MI/R), however, the protective cellular mechanism have yet to be revealed (Liu et al., 2009).

#### Invigorating and Replenishing Qi

##### Rhodiola

Rhodiola may relieve MI injury by improving angiogenesis in ischemic myocardium. Rhodiola up-regulated the expression of Flt-1 and Tie-2 in ischemic cardiac zone which are related to angiogenesis (Li et al., 2005). Flt-1, also known as VEGF receptor-1 (VEGFR-1) is a kind of tyrosine kinase. VEGF initiates the angiogenesis process via the activation of VEGFR-1 and VEGFR2 (Cudmore et al., 2012). Tie-2 is the receptor of angiopoietins. Angiopoietins-Ties axis are required for the formation of blood vessels (Hennings et al., 2016). A different study described in acute myocardial infarction rats, Radix et Rhizoma Rhodiolae Kirilowii may promote angiogenesis through stimulating HIF-1a, HIF-1β and VEGF. The expression of vWF, a marker of endothelial cells was also significantly increased (Gao et al., 2009).

##### Salidroside

Salidroside is an active compound extracted from Rhodiola and it has been reported that intramuscular administration of salidroside robustly enhanced blood perfusion recovery in hind-limb ischemia mice (Zhang J. et al., 2017). The beneficial effect of salidroside in peripheral artery disease may be extended to use in IHD and would need to be explored further.

##### Astragalosides

Astragaloside (AST), is the total saponin fraction isolated from *Astragalus membranaceus* (Fisch) (Wang et al., 2002) and has been used to develop drugs for therapeutics of cardiovascular diseases. AST promoted VEGF and bFGF expression which may contribute to increase in angiogenesis in rat models of myocardial infarction (Yu et al., 2015).

#### Other Active Components of Medicinal Plants

##### Berberine

Berberine is an alkaloid extracted from *Berberis and Berberis aristata* and has apparent preventive effects on cardiovascular diseases. Zhu et al. (2017) conducted the myocardial infarction (MI) surgery in mice fed with 100 mg/kg/day berberine. Two weeks after surgery, berberine significantly improved cardiac function and increased angiogenesis. Mice treated with berberine had increased expression of miR-29b. The protective effects of berberine were reversed by antagonism of miR-29b in human umbilical endothelial cells *in vitro* (Zhu et al., 2017). In contrast, reports suggest that miR-29b inhibits angiogenesis by targeting VEGF-A in endometrial carcinoma (Chen et al., 2017). MiR-29b also attenuated angiogenesis and tumorigenesis by directly targeting Akt3 (Li et al., 2017) or BCL2L2 (Chung et al., 2015). However, the role of miR-29b in angiogenesis may depend upon the cell type and pathophysiological setting such as ischemia.

##### Puerarin

Puerarin is a major effective ingredient extracted from the traditional Chinese medicine ge-gen (radix puerariae). Puerarin treatment (120 mg/kg, i.p.) reduced infarct size and promoted angiogenesis in the ischemic heart of rats with myocardial infarction. The cellular mechanism maybe via promotion of the expression of VEGF and eNOS (Zhang S. et al., 2006).

##### Extract of *Geum japonicum*

Li et al. (2006) isolated bioactivate fraction from Chinese herb *Geum japonicum*. *In vitro*, they screened and mixed the fractions that could stimulate angiogenesis and cardiomyogenesis. *In vivo*, the mixed fractions decreased infarct size, stimulate early development of new blood vessels and regenerate myocardium (Li et al., 2006).

### Chinese Herbal Formula

Chinese herbal formula has been reported to be effective in promoting angiogenesis in endothelial cells during intracerebral hemorrhage (REF). Therefore, whether Chinese herbal formula can have protective effects during IHD is yet to be established.

#### Danggui Buxue Tang (DBT)

Danggui Buxue Tang (DBT) is a classic Chinese herbal formula which consists of Astragali mongholici Radix and Angelica sinensis Radix (ASR). DBT may enhance angiogenesis and relieve endothelial dysfunction induced by lysophosphatidyl choline (Lin et al., 2017).

#### Xuefu Zhuyu Tang (XFZYT)

Xuefu Zhuyu Tang (XFZYT) is composed of peach kernel, safflower, angelica, chuanqiong, radix paeoniae rubra and other 11 kinds of natural compounds. XFZYT induced ECV304 endothelial cell proliferation, migration, and angiogenesis by up-regulating VEGF-VEGFR2 pathway. This could potentially explain how XFZYT may have the potential to promote angiogenesis in IHD (Gao et al., 2012).

#### Buyang Huanwu Tang (BYHWT)

Angiogenesis following intracerebral hemorrhage plays an essential role in maintaining brain functional and ultimately recovery. BYHWT treated mice exhibited increased vessels in the brain and enhanced VEGFR2 phosphorylation in brain micro vessels by PI3K/Akt signaling pathway (Cui et al., 2015).

### Chinese Patent Drugs

#### Shexiang Baoxin Pills

Shexiang Baoxin pills (SBPs) may partially participate in angiogenesis. Atherosclerosis and myocardial infarction rabbit models were established by Shen et al. (2010). High-fat diet accompanied with SBP was fed. Echocardiography results showed that SBP could improve cardiac functions. CD34 positive staining and increased VEGF and VEGFR-2 expressions indicated that SBP promoted angiogenesis (Shen et al., 2010). Another paper dived more deeply into the protective mechanism of SBPs. It may be mediated via up-regulation of 20-hydroxyeicosatetraenoic acid (20-HETE). HET0016 could inhibit the biosynthesis of 20-HETE and it could reverse the protective effects of SBPs on myocardial infarction. What’s more, SBP could promote circulating EPCs mobilization and VEGF expression (Huang et al., 2017).

#### QI-SHEN-YI-QI

QI-SHEN-YI-QI formula consists of astragalus, danshen, panax notoginseng, and dalbergia. Clinically, QI-SHEN-YI-QI formula was widely used to treat angina. Modern pharmacological studies show that QSYQ significantly decreased infarct area, and promoted microvessel density during 7 days in ischemic rats with left anterior descending artery (LAD) ligation. QSYQ treatment group induced the increased mRNA and proteins of VEGF, bFGF and PDGF-B (Zhang et al., 2010). In microvascular endothelial cells, Dai GH found that Qi-Shen-Yi-Qi Dripping Pills could promote the ECs migration and tube formation. miRNA chip and qPCR techniques were used to analyze the expression of miRNA and found that miR-223-3p was the target of QSYQ (Dai et al., 2016).

#### Tongxinluo (TXL)

Tongxinluo has the effects of promoting Qi, activating blood and dredging collaterals to relieve pain. Cardiac microvascular endothelial cells (CMECs) stimulated by Tongxinluo secreted more cytokines who were involved in CMECs proliferation, growth and differentiation, as well as chemotaxis and transport in ischemia/reperfusion injury. Among them, PDGF, bFGF, ANG-1, and VEGF all increased which means that Tongxinluo may be associated with angiogenesis (Cui et al., 2016).

#### Xuesetong Soft Capsules

Xuesetong soft capsules is composed of Notoginseng total Saponins. Wang et al. (2012) constructed the classical acute myocardial infarction rat models. Xuesetong soft capsules were fed for 6 weeks. Delightingly, Xuesetong soft capsules treatments could accelerate angiogenesis and VEGF mRNA expression in infarcted border zone (Wang et al., 2012).

## Conclusion

Ischemic heart disease is a serious threat to human health, the incidence is continuously increasing in conjunction with the prevalence of diabetes, metabolic disorders and the aging population. Therapeutic angiogenesis is a promising method in the management of IHD. Accumulating evidence in animal models has demonstrated that blood-activating and stasis-resolving Chinese herbal medicines have pro-angiogenic effects, the mechanisms of which are centered around the vascular growth factor signaling pathway. Nevertheless, further large scale blinded randomized clinical trials are essential to prove the effectiveness of the treatment and clarify our understanding of the therapeutic potential. If the traditional Chinese herbal medicines are to be used alongside contemporary therapy the safety and efficacy of therapeutic angiogenesis deserves more attention. Future studies will need to extend to dosing regime, delivery route and the safety of use alongside current drug therapeutics.

Chinese herbal medicines are apparently effective in the treatment of IHD because of their multi-targeting. However, the exact pro-angiogenic mechanism of Chinese herbal medicines still remains unclear and requires to be fully validated. Without these steps it makes it difficult to extend to the world. At present the understanding of the pro-angiogenic mechanism of Chinese herbal medicines is incomplete, and mainly focuses on the vascular growth factors. Deeper understanding and more specific signaling pathways need to be explored. For example, endothelial cells (ECs) display a highly migratory and proliferative state during vessel sprouting (Eelen et al., 2015). ECs metabolism are involved in angiogenesis (De Bock et al., 2013) and it may become a new candidate of Chinese herbal medicines. Inducing stem cells to secret vascular growth factors or form new vessels may be another candidate. In general, to explore the mechanism and effective molecular targets of Chinese herbal medicines are of importance, which will provide new insights into therapeutic angiogenesis and promote the modernization of Chinese herbal medicines.

## Author Contributions

DG, XC, and YW performed and wrote the manuscript. TL, SJ, and WW modified the manuscript. CM modified, edited, and contributed to the manuscript. All authors reviewed the manuscript.

## Conflict of Interest Statement

The authors declare that the research was conducted in the absence of any commercial or financial relationships that could be construed as a potential conflict of interest.

## Footnotes

1 https://www.ncbi.nlm.nih.gov/pubmed/
